# Effect of Heat Softening versus Ultrasonic Removal of Root-End Gutta-Percha on the Quality of Root-End Preparation for Endodontic Microsurgery

**DOI:** 10.1155/2021/8320234

**Published:** 2021-08-02

**Authors:** Zhiting Ling, Ziting Zheng, Yuting Zeng, Lifang Jiang, Yuan Wu, Buling Wu, Wenjuan Yan

**Affiliations:** Department of Conservative and Endodontic Dentistry, Nanfang Hospital, Southern Medical University, 1838 N Guangzhou Road, Guangzhou 510515, China

## Abstract

This study is aimed at comparing the efficacy of heat softening and ultrasonic removal of root-end gutta-percha during retrograde preparation for root apical microsurgery. Recently extracted single-rooted premolars (*n* = 40) were prepared with standardized endodontically treated and root-end resection and then randomly divided into four groups that received root-end cavity preparation using four different instruments: a diamond-coated ultrasonic tip (AS3D; SACTEON, Paris, France), AS3D with the modified plugger of cordless heat carrier (MSE; B&L Biotech, Bala Cynwyd, PA, USA), stainless steel ultrasonic tip (Jetip-2; B&L Biotech, Bala Cynwyd, PA, USA), or Jetip-2 with MSE. The time required for root-end preparation was recorded. The root apex samples were examined by scanning electron microscopy to assess root surface microcracks and marginal integrity. The remnants of gutta-percha on the cavity walls were evaluated using a stereomicroscope. Statistical analysis was performed using Student's *t*-test and Wilcoxon rank-sum test (*P* < 0.05). Usage of MSE with Jetip-2 significantly reduced preparation time from 99.8 ± 6.8 s to 32.4 ± 1.0 s (*P* = 0.009), the number of microcracks from 42 to 13 (*P* = 0.036), and the remnants of gutta-percha from 80% to 20% (*P* = 0.041). Similar results were observed for AS3D with MSE. The heat softening of MSE was effective in cleaning gutta-percha, thus greatly improving the efficiency of the root-end preparation, thereby producing a root-end cavity with fewer microcracks and better marginal integrity.

## 1. Introduction

Despite adequate endodontic treatment, failure may occur because of complex canal anatomy, apical cysts, and extraradicular infections [1]. Endodontic microsurgery is a reliable treatment approach with a success rate of 95.2% for teeth in which conventional endodontic treatment has failed [2–4]. It can facilitate the complete debridement of the root canal and ensure adequate sealing of the root canal apex.

The literature has highlighted the importance of root-end preparation in successful endodontic microsurgery [5, 6]. In recent years, ultrasonic tips have been gaining more extensive application than conventional rotary burs because of their unique advantages when used for root-end preparation [7, 8]. They allow safe access to the long axis of the root canal, decreasing the risk of lateral perforation and preserving an adequate environment for retrograde obturation. However, some studies have reported that apical microcracks appear during root-end preparation with ultrasonic tips [9–13], which increase the risk of apical leakage, reduce the mechanical properties of the root-end, and negatively affect the long-term outcomes of endodontic microsurgery [14].

Thermoplasticized gutta-percha obturation permits not only better adaptation to root canal surface irregularities but also the dense packing of gutta-percha inside the root canals, thus garnering increased popularity [15]. However, gutta-percha fillings are difficult to remove during retrograde cavity preparation, even when using ultrasonic tips. Moreover, clinical practice has shown that it takes a prolonged time to remove gutta-percha fillings, especially for teeth with large canals, which may increase the frequency of root surface microcracks [16].

**Figure 1:**
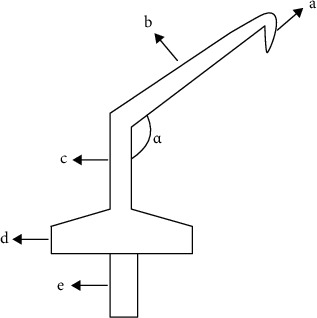
Sketch map of the modified plugger of heat carrier. (a) Working tip with a length of 3 mm and diameter 0.4 mm. (b) Prebent connector with a length of 15 mm. (c) Prebent connector 2 with a length of 6 mm. (d) Heat carrier connecting handle bayonet. (e) Cordless conductor. (*α*) 120-150°.

It is well known that gutta-percha can be easily manipulated with heated instruments. For example, SuperEndo B&L *α*^2^ (B&L Biotech, Bala Cynwyd, PA, USA), a cordless heat carrier specifically designed for the continuous condensation technique [17, 18], only takes 5 s to reach the temperature peak; consequently, it can efficiently remove the thermal-fill obturators with little potential harm to the surrounding tissues [19]. However, there are no heat carriers suitable for retrograde root canal preparation. Therefore, based on the system B heat source, we modified this plugger of a cordless heat carrier (Modified SuperEndo-*α*^2^, MSE; B&L Bala Cynwyd, PA, USA) according to the root-end ultrasonic tip, permitting easy manipulation in the apical region (Figure 1). In this study, the effects of heating, softening, and ultrasonic techniques were compared with respect to the preparation time, root surface microcracks, presence of gutta-percha, and marginal integrity of the root-end cavity for retrograde preparation.

## 2. Materials and Methods

This study was approved by the ethics committee of Nanfang Hospital, Southern Medical University (Guangzhou, China). Freshly extracted, single-rooted premolars that were free of caries, cracks, fractures, and root canal treatments were selected and stored in saline solution. All samples were confirmed to possess single canal systems using periapical radiographs. The working length was determined by subtracting 0.5 mm from the point at which the tip of a #10 K-file was visible at the apical foramen. Root canals were prepared up to X2 (ProTaper Next; Dentsply, Ballaigues, Switzerland) using a crown-down technique and irrigated with 2.5% sodium hypochlorite and 17% EDTA. After cleaning and shaping, each canal was dried and obturated with gutta-percha (Calamus Dual; Dentsply, Ballaigues, Switzerland) and AH Plus sealer (Dentsply DeTrey GmbH, Konstanz, Germany), applying the hot continuous-wave condensation technique. All teeth were then coronally sealed with glass ionomer cement (Fuji IX, GC Corp., Tokyo, Japan).

**Figure 2:**
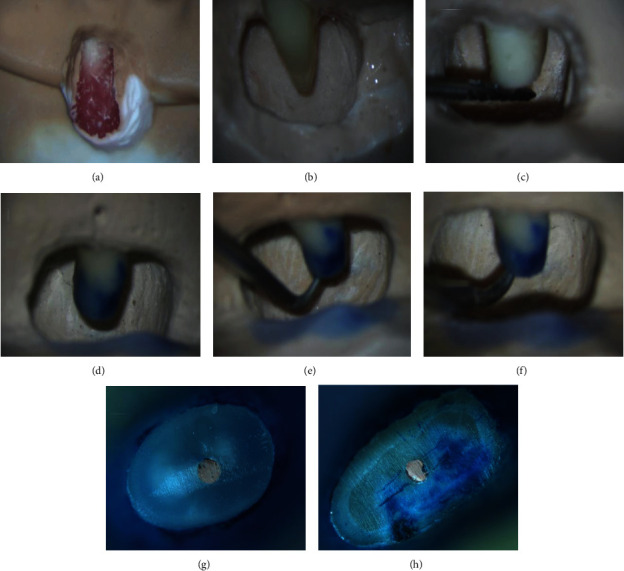
Simulated endodontic microsurgery in the premolar region. (a) Apically flap. (b) Curettage cyst. (c) Apical resected. (d) Apical staining. (e) Apical preparation. (f) Use the modified heat harrier for the removal of gutta-percha. (g) No apical surface microcracks after apicectomy. (h) Apical surface microcracks after apicectomy.

To mimic a periapical cyst, the root apices were surrounded by sticky wax with a volume of 1 × 0.5 × 0.5 cm and then embedded in dental plaster along the long axis of the premolar. Finally, the sticky wax was removed, exposing the root (Figures 2(a) and 2(b)). All procedures were performed by a single operator using a dental operating microscope (DOM; Carl Zeiss, Oberkochen, Germany). The apical 3 mm of each root was resected perpendicular to the long axis from the apex (Figure 2(c)) with H254LE (Komet, Gebr. Brasseler, Lemgo, Germany) carbide burrs. The resected root surfaces were coated with 1% methylene blue (Canal blue; Dentsply Sirona, Konstanz, Germany), dyed for 5 min, rinsed with water for 1 min, and checked under DOM at ×10 magnification to identify microcracks. Samples with root microcracks, as shown in Figure 2(h), were excluded from further investigation.

Forty teeth were randomly divided into four groups according to the instruments used for the root-end preparation: in group A, this was performed using a diamond-coated ultrasonic tip (AS3D; SACTEON, Paris, France); in group B, it was performed using AS3D with MSE (Figure 2(f)); group C was treated with a stainless ultrasonic tip (Jetip-2; B&L Biotech, Bala Cynwyd, PA, USA); and in group D, it was performed using Jetip-2 with MSE. The ultrasonic reverse preparation was performed using the tip of a P5 XS ultrasonic device (Satelec) set at the endo mode and a power setting of 6. The MSE was set to 180°C in the continuous mode. In groups B and D, the samples were first prepared with ultrasonic tips for 30 s, after which the MSE was used to soften and remove the root-end filling material. Root-end preparation was performed by applying intermittent and minimal pressure following the manufacturer's instructions until an apical cavity was achieved at a depth of 3 mm from the resected surface. The time required for preparation, from the onset of the procedure until no root filling material was visible on the cavity walls [8], was measured with a stopwatch under the DOM. All samples were taken out from the plaster model and placed under a stereomicroscope (SZX7; Olympus, Tokyo, Japan) at ×30 magnification to evaluate the presence of gutta-percha in the cavity [20] (Table 1(a)). The samples were then sputter-coated with gold, and the following characteristics were examined using a scanning electron microscope (SEM) at 10.00 kV, ×30 magnification, and low vacuum (S-4800N, Hitachi, Tokyo, Japan): (1) the number and type of root-end surface microcracks [21, 22] (Table 1(b)) and (2) the marginal integrity of the root-end cavity [7] (Table 1(c)).

Statistical analyses were performed using SPSS software (version 18.0; SPSS Inc., Chicago, IL, USA). The times required for root-end preparation were compared using Student's *t*-test. The Wilcoxon rank-sum test was performed to evaluate the differences among the four groups regarding root surface microcracks, marginal integrity, and the presence of gutta-percha. A *P* value of less than 0.05 was considered statistically significant.

## 3. Results

**Figure 3:**
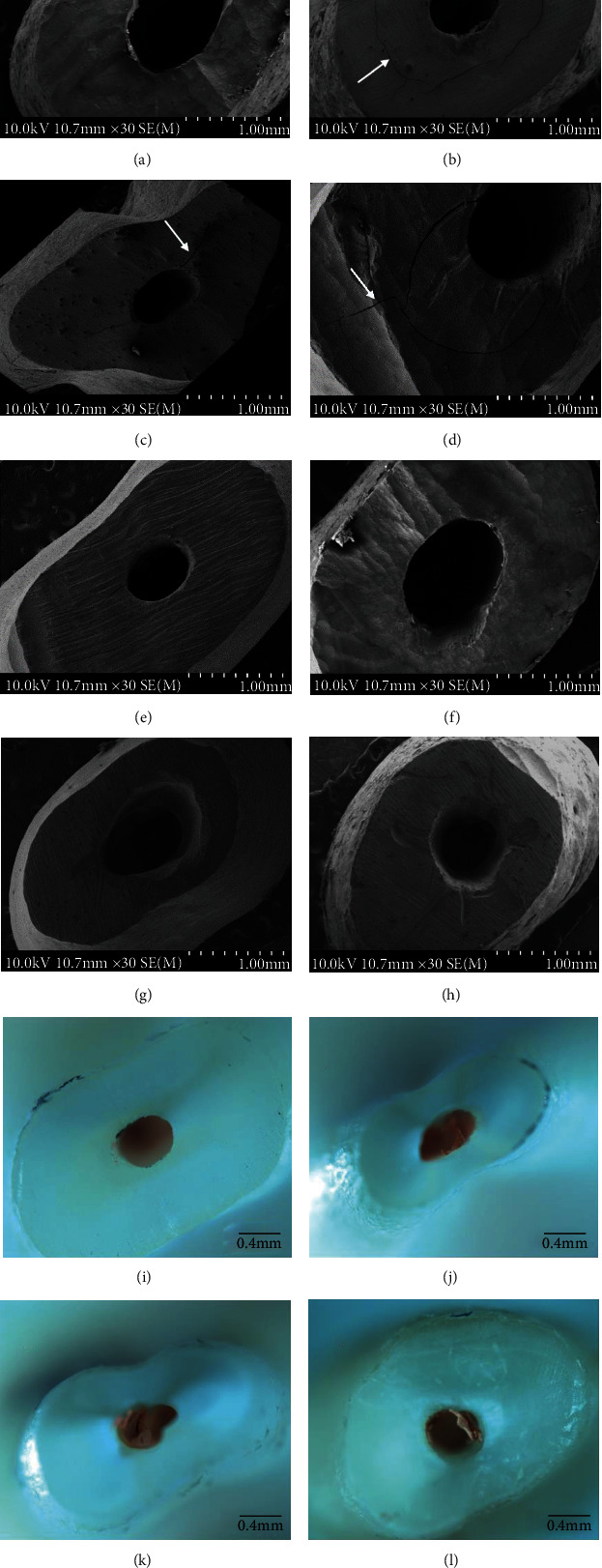
The typical morphology of root-end cavity preparation through SEM and stereomicroscope regarding root surface microcracks, marginal integrity of root-end cavities, and the presence of gutta-percha. (a) No microcracks. (b) Intradentinal microcracks. (c) Incomplete microcracks. (d) Complete microcracks. (e) Score 1: smooth and regular cavity. (f) Score 2: smooth and regular cavities with the presence of grooves on one or two walls. (g) Score 3: irregular cavities with the presence of grooves on three walls. (h) Score 4: irregular cavities with the presence of grooves on the four walls. (i) Type A: clean walls. (j) Type B: gutta-percha on one wall. (k) Type C: gutta-percha on two walls. (l) Type D: gutta-percha on three walls.

There was no significant difference in the time requirements and features of apical cavity preparation performed with the AS3D and Jetip-2 ultrasonic tips (Table 2). As shown in Table 3, the time of retrograde preparation using an ultrasonic tip (AS3D) with MSE was the shortest, but it was not significantly different from that using Jetip-2 with MSE (*P* > 0.05). However, a significant difference was observed between the techniques with and without MSE (*P* < 0.05). Regarding the number and types of root-end surface microcracks, significant differences were revealed between the retrograde preparation techniques with and without MSE (Table 3). The highest number of microcracks was recorded in both the AS3D and Jetip-2 groups. Most of the microcracks were incomplete cracks. Concerning the marginal integrity of the root-end cavity (Figures 3(e) and 3(h)), the maximum value of “4” was obtained in the AS3D and Jetip-2 groups, totaling 10.0% of the samples. The minimum score was “0” mostly observed in the groups of ultrasonic tips with MSE (70% of the samples from these groups). Significant differences (*P* < 0.05) were also revealed between the groups with and without MSE regarding marginal integrity (Table 4). Groups with MSE performed significantly better than the ultrasonic-alone preparation concerning gutta-percha removal (*P* < 0.05).

## 4. Discussion

Generally, during retrograde preparation of the root-end cavity, gutta-percha is removed using an ultrasonic technique, and it also poses the risk of microcrack formation. In the present study, the plugger of the heat carrier was modified to effectively remove the root-end gutta-percha and reduce the usage of ultrasonic tips, thus reducing the formation of microcracks.

To exclude the possibility of microcracks which resulted from root resecting and keep the sample integrality, the surface of the resected root was checked under DOM at ×10 magnification to identify microcracks. To exclude the influence of ultrasonic tools, two typical ultrasonic tips were chosen, and their performances were evaluated. The tip is covered with diamond particles, AS3D, as compared to the tip with microprojections integrated onto the main body, Jetip-2, which has greater resilience and wear resistance. The time required to prepare a retrograde root-end cavity, which is of major importance in clinical practice [13, 16, 23], was evaluated among groups with or without MSE. No significant difference was found in the mean time required for preparation using the AS3D and Jetip-2 ultrasonic tips, which agrees with previous studies that both diamond-coated tip and stainless steel tip present a similar cutting ability when preparing root-end cavities [12, 24]. In the groups that used MSE as an auxiliary instrument, the root-end preparation time was significantly shorter (*P* < 0.05). After the cavities were prepared with the ultrasonic tips, the MSE permitted the easy and rapid removal of the filling material in the root-end cavities. Furthermore, it delivered sufficient heat from the tip directly to the polymerized gutta-percha, thus allowing sufficient softening of the gutta-percha remnants inside the apical cavity, adaption of the irregularities of the intracanal anatomy, and achievement of a smoother gutta-percha section (Figure 3(i)). Therefore, groups of ultrasonic tips with MSE provide higher-quality root-end cavity walls. Previous studies found that higher bond strength values of root-end filling materials to dental walls could be achieved in higher-quality root-end cavity walls [25]. It is possible to improve the sealing ability of the root-end and reduce microleakage [25, 26]; this subject requires further study.

Root-end preparation is aimed at creating a well-defined root-end cavity without unnecessary damage to the tooth structure. One of the concerns during ultrasonic root-end preparation is the possibility of microcrack formation. In this experimental work, teeth that experienced cracks or fractures after root-end resection were excluded to avoid the illusion of preoperative cracking [22]. SEM analysis confirmed that the ultrasonic technique is always associated with the formation of microcracks, which is in agreement with the results of Peters et al. [9–13]. However, our results differ from those of Calzonetti et al. [23], in which ultrasonic root-end cavity preparation did not cause root microfractures by using an in situ impression technique, and other studies have also found no microcracks or fractures after root-end cavity preparation with ultrasonic tips [7, 27]. The difference between the results could be due to differences in the experimental conditions and the methods used to detect microcracks, suggesting that root-end microcrack detection may be a technique-sensitive process [10, 28–30]. A significantly lower incidence of microcrack formation was observed in the groups using the MSE (*P* = 0.001), probably because of the shorter root-end preparation time, thus reducing the incidence of cracks. Regarding the type of microcracks, most cracks were intradentinal or incomplete, which may be attributed to the low power setting of the ultrasonic unit applied for root-end preparation.

For the marginal integrity of the root-end cavity, the majority of the preparations (over 50%) showed apical cavities with irregular margins in the AS3D and Jetip-2 groups, which agrees with the results of Palma et al. [12]. Irregular margins may affect the sealing of root-end filling or favor bacterial retention, which may lead to long-term failure of the surgery because of the increasing risk of apical leakage [31].

Although the modified plugger demonstrated advantages in an *in vitro* retrograde root-end preparation, its applicability in clinical practice, as well as studies regarding the potential influence of periodontal tissue affected by heat, is yet to be confirmed.

## 5. Conclusions

The modified plugger of the cordless heat carrier demonstrated a promising benefit for root-end preparation, which reduced the operation time and increased the quality of the root-end cavity.

## Figures and Tables

**Table:** (a) Presence of gutta-percha. Adapted classification from Khabbaz et al. [20]

Designation	A	B	C	D	E
Description	Clean walls	Gutta-percha on 1 wall	Gutta-percha on 2 walls	Gutta-percha on 3 walls	Gutta-percha on 4 walls

**Table:** (b) Root-end surface microcracks. Adapted criteria proposed from Taschieri et al. [21] and Rainwater et al. [22]

	Number				Type		
Designation	A	B	C	D	I	II	III
Description	Absence of cracks	1 to 3 cracks	4 to 6 cracks	7 or more cracks	Intradentinal	Incomplete	Complete

**Table:** (c) Marginal integrity of root-end cavity. Adapted criteria proposed by Bernardes et al. [7]

Score	1	2	3	4
Description	Smooth and regular cavities	Smooth and regular cavities with presence of groove on 1 or 2 walls	Irregular cavities with presence of groove on 3 walls	Irregular cavities with presence of groove on 4 walls

**Table 2:** Results of the time requirements and features of apical cavity preparation performed with AS3D and Jetip-2 ultrasonic tips.

		AS3D	Jetip-2	*P*
Time		99.3 ± 9.3	99.8 ± 6.8	0.174
Number of microcracks	A	0	0	0.639
	B	6	7	
	C	4	3	
	D	0	0	
Type of microcracks	I	13	12	0.787
	II	19	20	
	III	13	10	
Cavity margin integrity	1	1	0	0.774
	2	4	5	
	3	4	4	
	4	1	1	
Presence of gutta-percha	A	1 (10%)	2 (20%)	0.912
	B	4 (40%)	4 (40%)	
	C	2 (20%)	2 (20%)	
	D	3 (30%)	2 (20%)	
	E	0	0	

**Table 3:** The time required for root-end preparation (seconds; mean ± standard deviation).

Groups	Time	*P*
AS3D	99.3 ± 9.3	<0.001
AS3D with MSE	33.2 ± 1.1	
Jetip-2	99.8 ± 6.8	0.009
Jetip-2 with MSE	32.4 ± 1.0	

**Table 4:** Results of the evaluation of root-end preparation.

		AS3D	AS3D+MSE	*P*	Jetip-2	Jetip-2+MSE	*P*
Number of microcracks	A	0	7	0.005	0	8	0.001
	B	6	2		7	1	
	C	4	1		3	1	
	D	0	0		0	0	
Type of microcracks	I	13	7	0.044	12	7	0.036
	II	19	4		20	6	
	III	13	0		10	0	
Cavity margin integrity	1	1	7	0.047	0	7	0.010
	2	4	2		5	1	
	3	4	1		4	2	
	4	1	0		1	0	
Presence of gutta-percha	A	1 (10%)	7 (70%)	0.037	2 (20%)	8 (80%)	0.041
	B	4 (40%)	2 (20%)		4 (40%)	2 (20%)	
	C	2 (20%)	1 (10%)		2 (20%)	0	
	D	3 (30%)	0		2 (20%)	0	
	E	0	0		0	0	

## Data Availability

The data used to support the findings of this study are included in this article.
